# Geographic Clustering of Polygenic Scores at Different Stages of the Life Course

**DOI:** 10.7758/rsf.2018.4.4.08

**Published:** 2018-04

**Authors:** BENJAMIN W. DOMINGUE, DAVID H. REHKOPF, DALTON CONLEY, JASON D. BOARDMAN

**Affiliations:** is assistant professor of education at Stanford University.; is assistant professor of medicine at the Stanford University School of Medicine.; is professor of sociology at Princeton University.; is professor in the Department of Sociology and director of the Health and Society Program in the Institute of Behavioral Science at the University of Colorado at Boulder

**Keywords:** polygenic scores, genetics, geography

## Abstract

We interrogate state-level clustering of polygenic scores at different points in the life course and variation in the association of mean polygenic scores in a respondent’s state of birth with corresponding phenotypes. The polygenic scores for height and smoking show the most state-level clustering (2 to 4 percent) with relatively little clustering observed for the other scores. However, even the small amounts of observed clustering are potentially meaningful. The state-mean polygenic score for educational attainment is strongly associated with an individual’s educational attainment net of that person’s polygenic score. The ecological clustering of polygenic scores may denote a new environmental factor in gene-environment research. We conclude by discussing possible mechanisms that underlie this association and the implications of our findings for social and genetic research.

The geographic clustering of specific genotypes could be an important biosocial pathway through which observed spatial correlations of morbidities and related health and social characteristics materialize (Kindig and Cheng 2013; However, such studies focused only on genetic Murray et al. 2006). Likewise, such potential Likewise, such potential clustering may complicate efforts to disentangle genetic from environmental influences with which they might covary. Early investigations into this issue did not find evidence for geographic clustering of genetic risk at the state level (Rehkopf, Domingue, and Cullen 2016). However, such studies focused only on genetic risks for physical health outcomes (coronary artery disease, diabetes, and ischemic stroke), quantified these genetic risks in a limited manner (using only genome-wide significant variants), and focused on the geographic concentration of genetic risk at birth. They thus did not allow for the potential dynamic of geographic mobility within a single generation, which may lead to an increase in the spatial patterning of genetic risk later in life. The testfor such clustering at different stages of the life course adds an important new dimension to the health and aging literatures.

It has long been known that genotypes are not distributed randomly across environments (Plomin, DeFries, and Loehlin 1977). Within this literature, some models of gene-environment interplay have incorporated a life course perspective, but there is as of yet little empirical evidence about specific traits and periods of the life course in which genotypes will become increasingly or decreasingly clustered in particular environments (Shanahan and Boardman 2009). The active gene-environment correlation (rGE) hypothesis is especially salient here as it suggests that people actively select into environments as a function of their genotype. We can evaluate the salience of this active rGE hypothesis by examining genetic clustering at the state level at different points of the life course—that is, when individuals have low degrees of autonomy to sort themselves genetically (childhood) and when they do have the agency and freedom to change environments (adulthood). Thus, evaluating whether life course-related, inter-state mobility is associated with changes in geographic concentration in genetics would provide critical information about migration in the gene-environment interplay paradigm.

This observation motivates the first question in this study: is there any evidence for the geographic clustering of genotypes, as operationalized by polygenic scores, at different points in the life course (Belsky and Israel 2014; Dudbridge 2016)?

## GENETICS AND THE LIFE COURSE

Life course research begins with the observation that individual development is a constant exchange between the specific characteristics of individuals and their social, physical, and cultural environments (Elder 1998). A large body of work has examined the concordance and discordance of behavioral and personality traits among very young twin pairs to estimate the extent to which genes contribute to specific traits. Two main observations emerge from this work. First, nearly all traits of interest to behavioral and social scientists—such as health, physical size, communication skills, cognitive ability, and behavioral disinhibition—are moderately influenced by genetics in which genes account for roughly one-third to one-half of their overall variation (Turkheimer 2000; Polderman et al. 2015). Second, the relative contribution of genes to many behavioral traits can change considerably over the life course. A particularly striking example of the latter is known as the Wilson effect (Bouchard 2013), which suggests that the heritability of cognitive ability increases as individuals age. The gene-environment typology anticipates such variation as a consequence of shifting environmental exposures. However, shifting environmental exposures may themselves be related to geno-type and such a possibility has important implications (Jaffee and Price 2007).

In the endeavor to understand the role of genetics in human behavior and well-being, a question of fundamental import is whether or not specific genetic polymorphisms affect complex traits similarly across different environments. Straightforward identification of gene-environment interactions (GxE) rests on the assumption that genes and environments are independent. Others have made clear that our ability to detect and understand GxE associations are seriously compromised in the face of rGE (Jaffee and Price 2007; Fletcher and Conley 2013). For example, early evidence suggested that sensitivity to stressful life events is conditioned by genotype. Individuals who have experienced the same stressful life event may have different mental health responses. Avshalom Caspi and colleagues suggest that some of this difference is due to the presence of the S’ allele in the 5HTTLPR locus, which is linked to serotonergic production and maintenance (2003). However, evidence indicates that carriers of this short allele may be more likely than others to be exposed to increased levels of stress or different types of stress (Risch et al. 2009). In that event, the proposed GxE interaction may be better characterized as an rGE association (Culverhouse et al. 2017). Such complications may help explain the mixed replication history for this finding and for GxE findings in the candidate gene literature more generally (Duncan and Keller 2011). To avoid this concern, researchers have made efforts to use environmental exposures that are most likely to be independent of genotype (Schmitz and Conley 2016; Domingue, Liu, Okbay, and Belsky 2017).

Others have used state of residence for these purposes arguing that selection of state of residence is unlikely to be driven by genetic factors—for example, smokers choosing a state of residence based on its pro-smoking features (Boardman 2009). With longitudinal data, we have some capacity for evaluating this claim. Specifically, we present statistical estimates that characterize the extent to which specific genetic polymorphisms linked to important outcomes are clustered across U.S. states. We pay particular attention to differences in these estimates at different stages of the life course.

## MECHANISMS RELATED TO GENE-ENVIRONMENT CORRELATION

We consider several potential mechanisms (active, passive, evocative, and mortality selection) through which gene-environment correlations come to be and how they may be related to the specific phenotypes we investigate as well as different periods in the life course. Active gene-environment correlations exist when individuals select into specific environments because of genetic polymorphisms that are linked to particular phenotypes and endophenotypes. Consider, for example, individuals for whom a healthy lifestyle—including the avoidance of tobacco products—is, in part, genetically influenced. Over time, it is possible that such individuals may select to live in states that provide a greater access to outdoor activities and other cardiovascular health-enhancing behaviors.

In contrast to active rGE, passive rGE is a situation in which children inherit their genes and their environments from their parents. This may simply reflect a form of population stratification, along the lines of what has been shown on a comparable geographic scale (Novembre et al. 2008; Nelis et al. 2009; Han et al. 2017). We hypothesize that the effect of passive rGE will be most pronounced when state of residence is measured at birth. Relatedly, the evocative rGE mechanism occurs when specific genotypes evoke specific environments. The most common example is that genetically oriented behaviors in childhood such as hostility or irritability may evoke more harsh parenting and educational environments for certain children (Jaffee and Price 2007). This model generally focuses on younger children who have limited capacity to select into environments but who may evoke certain environmental responses, such as from parents or teachers. As in regard to active and passive rGE, we again suspect that the effect will be most pronounced when measured at state of birth (though the role of evocative rGE may be limited in this study given the nature of phenotypes we consider). Although the evocative rGE model may be relevant to elderly populations when considering housing selection toward the end of life, the likelihood that one’s genetic characteristics would evoke selection into a specific state of residence seems implausible. We therefore focus on active and passive forms of rGE in our interpretation.

Finally, we consider a form of observed rGE that is rarely discussed in the rGE literature but that has special import given the nature of our sample of surviving older respondents (Zajacova and Burgard 2013). Our state-level estimates of average polygenic score (PGS) levels (and by extension, rGE and social genetic effects) are confounded by mortality selection in which the composition of those in the sample is increasingly the most healthy. Because the social and environmental characteristics of state or smaller places of residence may affect mortality, we could see greater state-level intra-class correlations (ICCs) later in the life course due to differential mortality associated with genotype (see Deaton and Lubotsky 2003).

## MECHANISMS RELATED TO ECOLOGICAL PENETRANCE

We consider the penetrance (association of genotype and phenotype) of the polygenic scores at both the individual and ecological (that is, state mean) level. Differences in penetrance between these two levels must be interpreted with care. We do not have the necessary data to make fine-grained distinctions about the mechanisms driving increased ecological penetrance and thus focus on asking about the operation of a single mechanism. Specifically, we ask whether penetrance has increased at the ecological level net of an individual’s genetic endowment. This could suggest that the genetic load of an individual’s within-state neighbors are predictive of an individual’s response, that is, social genetic effects, thus leading to a larger ecological than individual-level penetrance (Domingue and Belsky 2017). This mechanism would be potentially observable via the predictive power of the mean level of a polygenic score in the state net of an individual’s own polygenic score. However, a number of alternative mechanisms are also explanations for such a finding. The presence of direct environmental or GxE effects where the environmental influence is orthogonal to the effect of the mean polygenic score, attenuation due to measurement error, aggregation bias, and other nonlinearities could also drive increased ecological correlations. Uncertainty about the meaning of aggregate relationships would not be unique to this type of sociogenomic inquiry but may still provide insight into areas of GxE research that need proceed with caution (Mellor and Milyo 2001; Wilkinson 1996).

## DATA

We use data from the Health and Retirement Study (HRS). The HRS is a biennial survey of older Americans (age fifty and older), focusing on their health, family structure, and socioeconomic status. Due to the lack of comparability of genetic association results using the polygenic score approach across racial groups (Carlson et al. 2013; Martin et al. 2017), we focus on 8,629 respondents of European ancestry, as identified by their genetic data, born between 1905 and 1974 (mean = 1938, IQR = 1938–1946). We use behavioral, medical, and anthropometric measures.

### Measures

We describe the individual-level variables used in this study and provide their mean and standard deviation (SD) as operationalized here.

Alzheimer’s disease (M = 0.06, SD = 0.23): whether a respondent reported ever having memory-problems (waves 1–9) or Alzheimer’s (waves 10–11).

Body mass index (M = 29.7, SD = 6.0): maximum (Stokes and Preston 2016) over available waves.

Heart disease (M = 0.39, SD = 0.49): a binary indicator of whether a respondent ever reported heart disease.

Education (M = 13.2, SD = 2.5): total years of educational attainment.

Smoking (M = 0.57, SD = 0.50): an indicator of whether a respondent ever reported smoking.

Height (M = 1.7, SD = 0.1): maximum reported height.

Depression (M = 3.0, SD = 2.3): maximum number of Center for Epidemiological Studies-Depression (CESD) symptoms over all waves.

Arthritis (M = 0.75, SD = 0.43): an indicator of whether a respondent ever reports arthritis.

### States of Residence

Respondents’ state of residence at each wave of data collection is recorded as well as the state of birth and schooling for the respondent. Use of these geographic measures is complicated by the sampling scheme of the HRS. HRS employs a multistage sampling design. The first stage of sampling is metropolitan statistical areas (MSAs) or non-MSA U.S. counties. Current residents of states that contain MSAs or counties sampled by HRS may be represented in the HRS sample independently of where they were born. At the first wave of HRS data collection in 1992, respondents were in thirty-seven states plus the District of Columbia. We have a minimum of two respondents in a state and a maximum of 377 (mean = 105, SD = 76, IQR = 59–135). HRS respondents had to live in one of the MSAs at the time of data collection to be eligible for HRS, but many residents of these MSAs would have come from elsewhere in the country. As a consequence, the HRS sample was born across all fifty states and the District of Columbia. States have as few as one birth and as many as 646 (mean = 163, SD = 151, IQR = 40–240).

The sampling frame of U.S. MSAs and counties has two implications. First, people are sampled in a narrow geographic region in later life relative to where they were born. Thus, we anticipate more clustering later in life because of the geographic clustering induced by the sample design relative to at birth purely as a function of sample design. Furthermore, this restricts the generalizability of our findings in some respects since the HRS is not meant to be a representative sample at the state level. Second, not all states are represented in the baseline HRS survey, although HRS did sample from the most populous states, minimizing the negative implications for generalizability. To examine the degree to which our findings may fail to generalize due to the sampling scheme, we compared those who left their birth state at some point in the HRS to those who did not. The movers were heavier, had less education, and were more likely to have smoked. Thus, findings may be somewhat specific to the sample analyzed here.

### Genetic Data

Genetic data for the HRS is based on single nucleotide polymorphisms (SNPs) collected via two methods. The first phase was collected via buccal swabs in 2006 using the Quiagen Autopure method. The second phase used saliva samples collected in 2008 and extracted with Oragene. Genotype calls were then made based on a clustering of both data sets using the Illumina HumanOmni2.5–4v1 array (for a detailed report on the HRS genetic data, see Weir 2012). SNPs are removed if they are missing in more than 5 percent of cases, have low minor allele frequency (0.01), and are not in Hardy-Weinberg equilibrium (*p* < .001). We retain approximately 1.7 million SNPs after removing those that did not pass the quality control filters. We focus on nonHispanic whites for several reasons. First, allele frequency differences make direct comparisons of the distributions of polygenic scores across populations impossible. Second, due to differences in patterns of linkage, GenomeWide Association Study (GWAS) results discovered in European samples may not replicate in nonuropean samples (Carlson et al. 2013) and scores constructed from such results will perform differently out of sample (Martin et al. 2017). Third, the non-white sample of genotyped HRS respondents shows substantial selection relative to the non-Hispanic white sample (Domingue, Belsky, Harrati, Conley, Weir, and Boardman 2017).

## POLYGENIC SCORE CONSTRUCTION

We constructed polygenic scores (PGS) using published GWAS results. We computed scores for Alzheimer’s (Lambert et al. 2013), BMI (Locke et al. 2015), educational attainment (Okbay et al. 2016), cardiovascular disease (Schunkert et al. 2011), smoking (Tobacco and Genetics Consortium 2010), height (Wood et al. 2014), major depressive disorder (Ripke et al. 2013), and rheumatoid arthritis (Okada et al. 2014). These were selected to cover a range of health, anthropometric, and behavioral outcomes. Briefly, polygenic scoring was done with the PLINK software (Chang et al. 2015) using a previously discussed pipeline (Conley et al. 2016). SNPs in the HRS genetic database were matched to SNPs with reported results in a GWAS. For each SNP, a loading was calculated as the number of phenotype-associated alleles multiplied by the effect-size estimated in the original GWAS. Loadings were summed across SNPs to calculate the polygenic score. Scores were first residualized on the top ten PCs computed only among the non-Hispanic white respondents of HRS and then standardized to have a mean of zero and standard deviation of one for analysis for ease of interpretation.

## MODELING OF GENOTYPE AND PHENOTYPE CLUSTERING

Our analytic strategy for the detection of genetic clustering involves models of the form

(1) $$G_{is}\;=\;\alpha \;+\;u_{s\;}\;+\;e_{is},$$

where *G*_*is*_ is the polygenic score for individual in the *s-*th state. Most importantly, *e*_*is*_ captures the individual-level error term and *u*_*s*_ is a state-specific random intercept (capturing either state of birth or state of current residence). We assume that *u*_*s*_ ~Normal(0, $\sigma_{u}^{2}$ ) and then consider

(2) $$\text{ICC }=\;\frac{\sigma_{u}^{2}}{\sigma_{u}^{2}\;+\sigma_{e}^{2}}.$$

This quantity, the state-level ICC coefficient, is our key index of genetic concentration. That is, the contribution of $\sigma_{u}^{2}$ to overall variation in polygenic risk ($\sigma_{u}^{2}$ + 2 $\sigma_{e}^{2}$) is summarized as a ratio that simply describes the proportion of genetic variance nested within states.

## MODELS FOR PENETRANCE

To further evaluate the extent to which states matter for the clustering of specific phenotypes and their corresponding PGS values, we first compare individual-level correlations between each trait and the PGS for that trait with ecological correlations (for example, average state-level education with average state-level PGS) focusing on *state at birth*. Instances in which the ecological correlation exceeds what we would expect based on the individual-level correlation provide further support for importance of gene-environment interplay. We then present results in which we model the individual and ecological contributions of PGS to individual phenotype. For outcome *y*_*is*_ (where individual *i* is born in state *s*), we consider

(3) $$y_{is\;}\;=\;\alpha \;+\;b_{1}g_{is}\;+\;b_{2}{\bar{g}}_{s}\;+\;u_{s\;}\;+\text{ controls }+\;\varepsilon_{is}.$$

We consider both individual-level PGS (*g*_*is*_) and state-average PGS value (*g̅*_*s*_) to evaluate contributions of average genotype to state-level variation in each phenotype net of individual genetic endowments. Standard errors are corrected for state-level clustering (Zeileis 2004). We include demographic covariates (sex and birth year) as controls.

We also consider two sensitivity analyses related to equation (3). First, we estimate equation (3) in decennial birth cohorts to ensure that mortality selection and the changing salience of educational attainment are not driving our findings. Second, we further explore mortality selection via the use of weights previously discussed (Domingue, Belsky, Harrati, Conley, Weir, and Boardman 2017). These weights predict mortality prior to the genotyping window in HRS based on year of birth and a number of health conditions as well as educational attainment. We then use them as inverse probability weights to consider the sen sitivity of key findings to the fact that the HRS genetic data does not contain information on respondents who died prior to 2006 (Cole and Hernán 2008).

## STATE-LEVEL CLUSTERING OF PHENOT YPES

**Figure 1.**
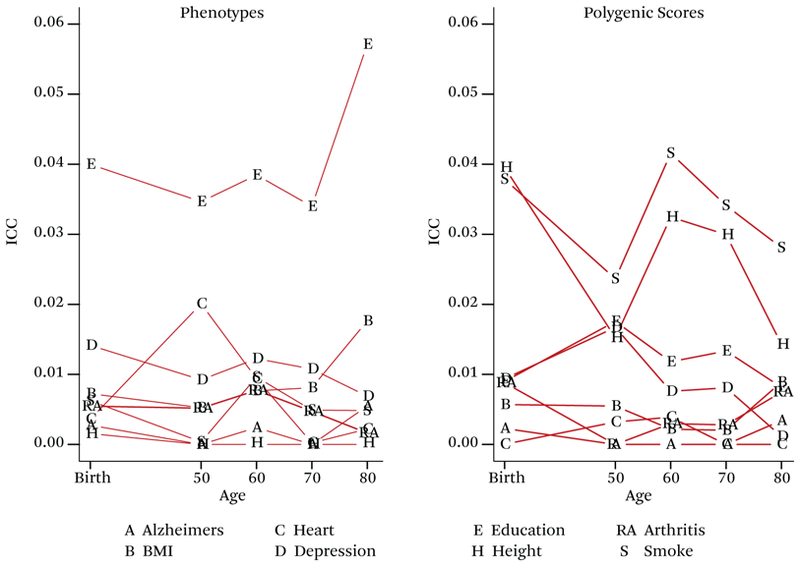
State-Level Clustering and Their Corresponding Polygenic Scores Across the Life Course *Source*: Author’s calculations based on HRS Rand files and genetic data (Weir 2012).

We first consider the state-level clustering of the phenotypes to establish benchmarks for interpreting the statelevel genetic concentrations. The left panel of figure 1 summarizes state-level clustering for each trait at birth and then in later life. As described, these estimates characterize the proportion of variation for each trait that is due to clustering at the state level. An ICC of zero would indicate identical average education scores across all states (that is, all the variation occurs within states) and an ICC of one would indicate that there was no individual variation within states. In our analysis, the overall contribution of state of residence and state of birth are relatively small for all of the traits that we examine (for example, ICCs < 5 percent) but the magnitude of these ICCs are in line with other work in this area (Mehta and Chang 2009).

Education is a clear outlier in having statelevel ICC values that are considerably higher than the other traits at all points of the life course. Differences are clear in resources (such as tax levels to support education), structures (such as city, county, and state differences in the governance and support of districts), and opportunities (such as labor demands for different levels of skills) that would translate to observable differences across states. Education is also the only trait that shows a substantial increase in state clustering across the latter part of the life course. We note two potential explanations. First, it may indicate that states with higher average levels of education also have lower mortality rates, and the composition of those with more education becomes more pronounced in certain states as a result. This is particularly important given the increasing levels of morbidity and mortality among middle-aged white adults in the United States (Case and Deaton 2015). Second, it could be due to migration associated with retirement. Both of these processes could in fact be acting in tandem to drive this increase.

## STATE-LEVEL CLUSTERING OF POLYGENIC SCORES (RGE)

We now consider one of the primary goals of this paper: to evaluate the degree of PGS clustering at the state level. The right side of figure 1 characterizes these magnitudes and how they change as a function of when in the life course state of residence is measured. We observe the largest clustering for the height and smoking polygenic scores. For these scores, observed clustering is higher than for any phenotype other than education. For education, we observe between 1 and 2 percent of the overall variation in the score to be clustered within state at any point in the life course. Next we explore the potential relevance of this clustering.

## ECOLOGICAL VERSUS INDIVIDUAL PENETRANCE

**Figure 2.**
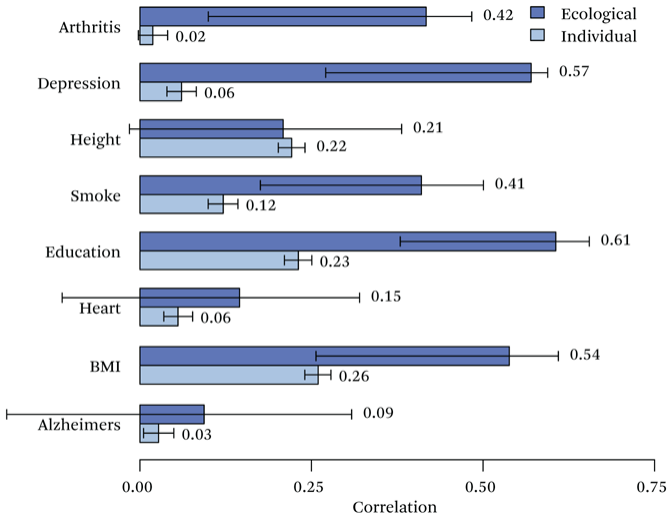
Correlations Between Polygenic Risk Scores and Their Corresponding Traits at Individual and Ecological Levels *Source*: Author’s calculations based on HRS Rand files and genetic data (Weir 2012).

We now turn to considerations of penetrance at the individual and ecological level *based on state of birth*. By comparing the individual and ecological correlations, we can provide indirect evidence for potential environmental enhancement of rGE through mechanisms that are generally, and perhaps incorrectly, characterized as GxE associations. In figure 2, the light gray bars focus on the correlation of individual phenotypes and PGSs. At the individual level, the largest observed association is (r = .26) is for BMI followed by education (r = .23) and height (r = .22). The darker bars in this figure depict state-level ecological correlations. Consider first height. The individual and ecological correlations are roughly comparable, suggesting a situation in which the translation of height-related genetics to physical stature is an individual-level phenomenon. This is perhaps intuitive given our understanding of physical growth as a largely within-person phenomenon.

But the story is quite different for depression, smoking, and education. In these cases, ecological correlations are larger than the individual correlations. This suggests the possible existence of higher-order process through which environmental differences (such as the mean genetic endowment within a state) are moderating the genotype-phenotype association when considered at higher levels (other explanations are also possible, we return to these in discussion). Consider the ecological correlation between the state mean education and the associated PGS (r = 0.61). This is 2.7 times the individual-level correlation and provides clear evidence that there is something additional of interest occurring in the context of states. To further interrogate this possibility, we examine analyses in which we predict individual phenotype using both individual and state-level genotype.

**Figure 3.**
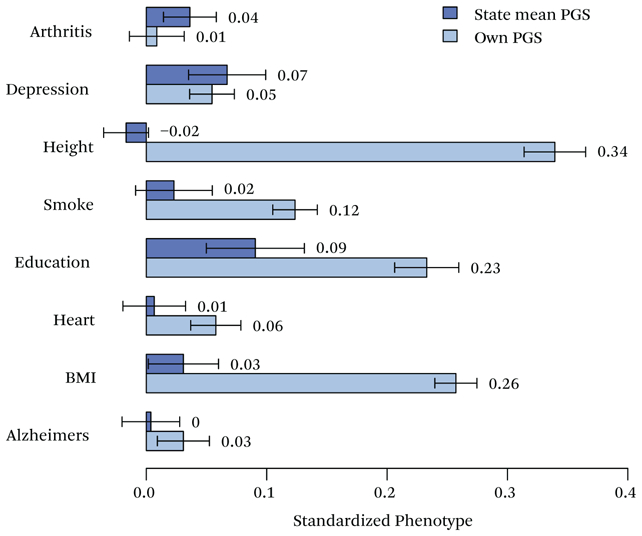
Standardized Multilevel Regression Estimates for the Effect of Each PGS *Source*: Author’s calculations based on HRS Rand files and genetic data (Weir 2012).

In figure 3, we consider estimates from equation (3). Returning to height, as expected, the state-level PGS did not offer any predictive power net of individual PGS. We observe similar results for smoking, heart disease and BMI, suggesting that, for these phenotypes, little residual information is left in the state-mean polygenic score. However, we observe markedly different findings for depression and educational attainment. For these phenotypes, the state-mean PGS predicts net of one’s polygenic score. We conducted two additional sensitivity analyses. First, we adjusted results for mortality selection prior to genotyping. Results were comparable; after weighting, the coefficient for state-level PGS mean was 0.08 (se = 0.012) for educational attainment and 0.08 (se = 0.012) for depression in their respective analyses. Second, we considered analyses for education restricted to the birth cohorts of the 1930s and 1940s to determine how sensitive results were to the changing salience of education over the years represented in the HRS birth cohorts. Again, findings were largely consistent. For 1930–1939 births, we estimated a coefficient of 0.06 (se = 0.020) for the mean educational attainment PGS. For 1940 to 1949 births, the respective estimate was 0.10 (se = 0.021). This allows for the possibility of a crucial role being played by the environment in determining how quantities of human capital develop; that is, these phenomena may have important between-person mechanisms.

## DISCUSSION

This study focuses on the potential for geographic clustering and moderation of genetic effects across a number of outcomes important for both mental and physical health. Polygenic scores demonstrate different magnitudes of clustering with most scores showing relatively little clustering. These results are important for research in gene-environment interaction research because the environment is often believed to be independent of genotype. Earlier work relied on the assumption that the state of residence was unlikely to be associated with specific genetic polymorphisms associated with specific genetic polymorphism. Thus, states were ideal candidates for the study of GxE given their relative exogeneity (Boardman 2009). This work noted the potential implications of rGE between specific polymorphisms and state of residence, but was unable to test this assumption given the lack of appropriate molecular data at that time.

Here, we are able to provide estimates about the likelihood of this type of selection bias. This clustering is indeed small, but also similar to the observed phenotypic clustering in many cases. Genes related to smoking were among the most concentrated. It is unclear whether this selection affects the previously reported GxE results at the state level (Boardman 2009). Even the small amounts of genetic clustering observed in figure 1 may be substantively important depending on the genetic penetrance for that phenotype (for example, weak genetic concentration for a highly penetrant phenotype might be of interest).

Indeed, there do seem to be occasions in our data in which relatively weak geographic PGS concentrations lead to provocative associations. In particular, we observe cases where ecological correlations are substantially larger than individual correlations. Moreover, for depression and education, we have evidence to suggest that the statelevel mean polygenic score for these traits is predictive of the trait net of an individual’s own genetic endowment. This might be so for a number of reasons. One set of explanations is mechanical. For example, misspecification at the individual level (such as nonlinearity in the penetrance of the PGS or measurement error) or even the nonrepresentative geographic sampling scheme of the HRS may lead to the inflated ecological correlations

An alternative explanation has to do with the nature of the phenotype. Although we cannot rule out mechanical reasons for our observations in figure 3, one key distinction merits attention. Educational attainment is a social phenotype. The very act of accruing years of education is a process that is typically provided by society and co-occurs with one’s age peers. These facts may help explain our findings in a number of ways. Having neighbors more inclined themselves toward education may bolster the effects of existing public education infrastructure because of greater support, via increased funding, for example, of educational programs. Such a mechanism is potentially related to the dual inheritance of genes and culture (Cavalli-Sforza and Feldman 1981). To the extent that the educational PGS merely reflects subtle genetic stratification that itself is correlated with cultural (or other environmental) conditions associated with educational attainment, what we report here could be confounded. Previous work uses sibling models to show the robustness of the PGS within families (after breaking any ancestry-PGS confounding) so at least some evidence suggests that the influence of confounding should be relatively limited (Domingue et al. 2015; Conley et al. 2015; Rietveld et al. 2014). That said, confidence in a lack of confounding at the individual level may not easily translate to the aggregate level. Social mechanisms also have a role in the etiology of depression, but research on the extent to which the results from that GWAS are susceptible to confounding is scant (Thoits 1995).

Findings related to the educational attainment polygenic score are consistent with the existence of “social genetic effects” but not dispositive (Domingue and Belsky 2017; Baud et al. 2017; Rauscher, Conley, and Siegal 2015). Identification of compositional effects is challenging (Angrist 2014). As in earlier work, we rely on cross-phenotype comparisons to guide interpretation (Cohen-Cole and Fletcher 2008). In particular, we note a clear distinction be tween educational attainment and height-BMI. Findings observed here are similar to those observed in another context in which the educational attainment PGS of schoolmates is associated with educational attainment (Domingue, Belsky, Fletcher, Conley, Boardman, and Mullan Harris 2017). In contrast, the genetics of school peers related to BMI and height were not predictive of phenotype. More research is needed to isolate the specific mechanism driving these findings and to tease out implications for spatial differences in education. This said, our findings raise questions about the extent to which educational attainment and BMI or height are phenotypes that are exchangeable in biologically informed analyses, such as a GWAS.

Our analysis has limitations. The primary limitation has to do with the nature of data available in the HRS. Given the nature of the HRS sampling, the geographic data is not fully representative. Specifically, because the HRS samples counties, it may be that all the respondents from a state are drawn from a relatively urban county that does not reflect the diversity of residential experiences within the state. This limits our ability to understand anything about levels for a particular state, and whether differential migration or other mechanisms of selection occur more strongly at finer levels of geography. U.S. metropolitan areas are a natural candidate for examination given the recent interest in smaller area mortality rates (Chetty et al. 2016). Finally, the phenotypes considered here are not clinical phenotypes and presumably contain measurement error.
